# Aminoguanidine Protects Boar Spermatozoa against the Deleterious Effects of Oxidative Stress

**DOI:** 10.3390/pharmaceutics10040212

**Published:** 2018-11-01

**Authors:** Eliana Pintus, Martin Kadlec, Marija Jovičić, Markéta Sedmíková, José Luis Ros-Santaella

**Affiliations:** Department of Veterinary Sciences, Faculty of Agrobiology, Food and Natural Resources, Czech University of Life Sciences Prague, Kamýcká 129, 165 00 Praha 6-Suchdol, Czech Republic; eliana.pintus27@gmail.com (E.P.); ma.kadlec13@email.cz (M.K.); jovicic@af.czu.cz (M.J.); sedmikova@af.czu.cz (M.S.)

**Keywords:** antioxidant capacity, lipid peroxidation, nitric oxide, oxidative stress, sperm velocity

## Abstract

Aminoguanidine is a selective inhibitor of the inducible nitric oxide synthase (iNOS) and a scavenger of reactive oxygen species (ROS). Numerous studies have shown the antioxidant properties of aminoguanidine in several cell lines, but the in vitro effects of this compound on spermatozoa under oxidative stress are unknown. In this study, we tested the hypothesis that aminoguanidine may protect against the detrimental effects of oxidative stress in boar spermatozoa. For this purpose, sperm samples were incubated with a ROS generating system (Fe^2+^/ascorbate) with or without aminoguanidine supplementation (10, 1, and 0.1 mM). Our results show that aminoguanidine has powerful antioxidant capacity and protects boar spermatozoa against the deleterious effects of oxidative stress. After 2 h and 3.5 h of sperm incubation, the samples treated with aminoguanidine showed a significant increase in sperm velocity, plasma membrane and acrosome integrity together with a reduced lipid peroxidation in comparison with control samples (*p* < 0.001). Interestingly, except for the levels of malondialdehyde, the samples treated with 1 mM aminoguanidine did not differ or showed better performance than control samples without Fe^2+^/ascorbate. The results from this study provide new insights into the application of aminoguanidine as an in vitro therapeutic agent against the detrimental effects of oxidative stress in semen samples.

## 1. Introduction

Oxidative stress arises when the production of the reactive oxygen species (ROS) overwhelms the intrinsic antioxidant defense of a biological system, leading to cell damage and death [1]. As a result of their metabolic activity, cells normally produce ROS, which are also required at certain levels for processes, such as cell signaling, mitochondrial function, and immune response [2,3,4]. Several factors (e.g., age, cigarette smoke, and ionizing radiation) and pathological conditions (e.g., cancer, diabetes, and infections) can also increase the amount of ROS to be above physiological levels, leading to oxidative stress. Across cells, spermatozoa are particularly susceptible to the damage caused by oxidative stress due to the high content of polyunsaturated fatty acids in their membranes and their limited antioxidant defence [5,6,7]. Despite certain levels of ROS being required for normal sperm function, their overproduction (due to pathological conditions, semen handling and storage) is detrimental for male fertility both in humans [8,9] and domestic animals [6,10].

Nitric oxide (NO**^•^**) is a short-living gas and a free radical that participates in many physiological (e.g., immune response, regulation of vascular tone and permeability) and pathological (e.g., cancer and neurological diseases) processes [11,12,13,14]. In the male reproductive system, NO**^•^** contributes to penile erection, sperm motility, capacitation, hyperactivation, and acrosome reaction [15,16]. In biological systems, NO**^•^** can be generated through non-enzymatic pathways by either direct disproportionation or reduction of nitrite under acidic and highly reduced conditions [17]. However, NO**^•^** is mainly synthesized from L-arginine by three NO synthase (NOS) isoforms: Neuronal (nNOS), endothelial (eNOS), and inducible (iNOS). All isoforms play a major role in the control of reproductive processes [18] and are expressed in human, mouse, and boar spermatozoa [19,20,21] among others. Unlike the other isoforms, iNOS is calcium independent and generates a large amount of NO**^•^** over prolonged periods (from seconds to days) [18]. Moreover, the iNOS isoform is expressed during inflammation or infection in activated leukocytes [13], which are the main source of ROS in the semen together with abnormal spermatozoa [22,23]. Therefore, the inhibition of the iNOS isoform may contribute by protecting against the detrimental effects of the oxidative stress in the semen.

Aminoguanidine is a selective inhibitor of the iNOS isoform [24] and a scavenger of hydrogen peroxide (H_2_O_2_), hypochlorous acid (HOCl), hydroxyl (**^•^**OH) and peroxynitrite (ONOO**^•^**) radicals [25]. Moreover, aminoguanidine was the first inhibitor of the advanced glycation pathway [26] with similar effects to those of the polyamines, spermine, and spermidine, which are abundant in sperm samples [27]. In a recent study conducted by our research group [28], we found that aminoguanidine improves some sperm kinetic parameters during boar semen storage at 17 °C. Moreover, aminoguanidine protects against the negative effects of oxidative stress induced by environmental pollutants [29] and pathological conditions, such as varicocele [30,31,32,33]. Nonetheless, the in vitro effects of aminoguanidine on sperm cells under induced oxidative stress are still unknown.

The aim of this study was to evaluate the in vitro effects of aminoguanidine on sperm cells under induced oxidative stress. Due to its powerful antioxidant activity, we hypothesized that aminoguanidine may protect against the deleterious effects of oxidative stress in sperm samples. To test our hypothesis, sperm samples were treated with Fe^2+^/ascorbate, which induces lipid peroxidation by catalyzing the production of **^•^**OH, the most potent free radical known [34]. The total antioxidant capacity, lipid peroxidation, sperm kinetics, plasma membrane integrity, and acrosomal status were evaluated in samples treated with aminoguanidine (10, 1, and 0.1 mM) and compared to those of control samples with or without oxidative stress. The results from this study indicate that aminoguanidine could be used as an efficient in vitro therapeutic agent for the treatment of sperm disorders associated with oxidative stress.

## 2. Materials and Methods

Reagents were purchased from Sigma-Aldrich (Prague, Czech Republic) unless otherwise stated.

### 2.1. Collection and Processing of Sperm Samples

Commercial sperm doses from 15 boars of different breeds (i.e., Czech Landrace, Czech Large White, Pietrain, Duroc, and Přeštice Black-Pied) and hybrid genetic lines were purchased from a breeding company (Chovservis, Hradec Králové, Czech Republic). Sperm-rich fractions were collected by the gloved-hand method, diluted with Solusem^®^ extender (AIM Worldwide, Vught, Netherlands), and transported to the laboratory at 17 °C. Only sperm samples with at least 75% motile spermatozoa were used for these experiments. To reduce the effect of male variability, equal volumes of sperm doses from three boars were mixed for each replicate. After this, sperm concentration was checked using a Bürker chamber and samples were further diluted with Solusem^®^ to get a final concentration of 20 × 10^6^ spermatozoa/mL. The samples were then randomly allocated into five groups: Control (CTR), control under oxidative stress (CTR-ox), and three treatments of aminoguanidine under oxidative stress (10, 1, and 0.1 mM, respectively). Aminoguanidine was freshly prepared on the day of the experiment (stock solution: 0.2 M) by dissolving aminoguanidine hydrochloride in phosphate buffered saline (PBS) and diluted with sperm samples to give a final concentration of 10, 1, and 0.1 mM. For CTR-ox samples, an equal volume of PBS solution was added. Oxidative stress was induced by 0.05 mM FeSO_4_ and 0.5 mM sodium ascorbate (Fe^2+^/ascorbate), a ROS generating system that is specific for inducing lipid peroxidation [35]. The experiment was replicated five times using five different semen pools. All sperm analyses were performed at 0 h (after 20 min of incubation, control only), 2 h, and 3.5 h of incubation in a water bath at 38 °C (Supplementary Dataset).

### 2.2. Assessment of Total Antioxidant Capacity

At the end of each incubation time, 300 μL of each sample was centrifuged at 2000× *g* for 10 min. After this, 150 μL of supernatant was collected and stored at −80 °C until analysis. The total antioxidant capacity was determined by spectrophotometry (Libra S22, Biochrom, Harvard Bioscience Company, Cambridge, UK) at 660 nm using the method described by Erel [36]. A standard curve was established using the known concentrations of 6-hydroxy-2,5,7,8-tetramethylchroman-2-carboxylic acid (Trolox). The total antioxidant capacity was expressed as mM Trolox equivalents. This assay was run in duplicate for each sample.

### 2.3. Assessment of Sperm Motility

A sperm aliquot (5 µL) was loaded into a pre-warmed (38 °C) Spermtrack chamber (PROiSER R + D S.L., Paterna, Spain; chamber depth: 20 µm). Sperm motility was evaluated subjectively by estimating the percentage of motile spermatozoa to the nearest 5% and the quality of movement (QM) using a scale from 0 (lowest: No motility) to 5 (highest: Progressive and vigorous movements). The sperm motility index (SMI) was calculated according to the following formula: [% individual motility + (QM × 20)]/2. Sperm kinetics were assessed by Computer Assisted Sperm Analysis (CASA; NIS-Elements, Nikon, Tokyo, Japan and Laboratory Imaging, Prague, Czech Republic), which consists of an Eclipse E600 tri-ocular phase contrast microscope (Nikon, Tokyo, Japan), equipped with a 10× negative phase-contrast objective (Nikon, Tokyo, Japan), a warming stage set at 38 °C (Tokai Hit, Shizuoka, Japan), and a DMK 23UM021 digital camera (The Imaging Source, Bremen, Germany). A total of nine descriptors of sperm kinetics were recorded after analyzing six random fields: Total motility (TM, %), progressive motility (PM, %), average path velocity (VAP, µm/s), curvilinear velocity (VCL, µm/s), straight-line velocity (VSL, µm/s), amplitude of lateral head displacement (ALH, μm), beat-cross frequency (BCF, Hz), linearity (LIN, %), and straightness (STR, %). The standard parameter settings were as follows: Frames per second, 60; minimum of frames acquired, 31; VAP ≥ 10 μm/s to classify a spermatozoon as motile; and STR ≥ 80% to classify a spermatozoon as progressive. A minimum of 200 motile sperm cells were analyzed per sample.

### 2.4. Assessment of Lipid Peroxidation

Lipid peroxidation was assessed using the thiobarbituric acid reactive substances (TBARS) assay as previously described [35,37]. At the end of each incubation time, sperm aliquots were collected and stored at −80 °C until analysis. The absorbance of the sample was measured by spectrophotometry at 532 nm. A standard curve was established using the known concentrations of 1,1,3,3-tetramethoxypropane (malondialdehyde, MDA). The levels of lipid peroxidation are shown as µmol of MDA per 10^8^ spermatozoa. This assay was run in duplicate for each sample.

### 2.5. Assessment of Sperm Plasma Membrane Integrity

The assessment of head membrane integrity was performed, as previously described [38,39]. Briefly, sperm samples were incubated with carboxyfluorescein diacetate (stock solution: 0.46 mg/mL in dimethyl sulfoxide), propidium iodide (stock solution: 0.5 mg/mL in PBS), and formaldehyde solution (0.3%) for 10 min at 37 °C in the dark. After this, 200 spermatozoa were evaluated in each sample using epi-fluorescence microscopy (40× objective) and the sperm cells showing complete green fluorescence over the head were considered to have an intact head membrane. The tail membrane integrity was determined using the hypoosmotic swelling test as previously described [39,40]. Briefly, sperm samples were diluted into a pre-warmed hypoosmotic solution (7.35 g/L sodium citrate and 13.51 g/L fructose) and incubated for 30 min at 38 °C. At the end of the incubation, 200 spermatozoa were evaluated using phase-contrast microscopy (40× objective) and the sperm cells showing swollen tails were considered to have an intact tail membrane.

### 2.6. Assessment of Acrosomal Status

In order to determine the percentage of sperm cells with a normal apical ridge (NAR) [41], the samples were fixed in 2% glutaraldehyde solution and examined under phase contrast microscopy (40× objective). Two-hundred spermatozoa were evaluated for each sample. The percentage of damaged acrosomes was determined according to the protocol described by García-Vázquez et al. [42]. Briefly, sperm samples were smeared onto glass slides, air-dried, and fixed with methanol for 10 min at room temperature. After this, samples were washed twice with PBS and incubated with peanut agglutinin-fluorescein isothiocyanate (PNA-FITC, stock solution: 0.2 mg/mL in PBS) for 10 min at 37 °C in the dark. Finally, the samples were washed for 5 min with PBS and evaluated under epi-fluorescence microscopy (40× objective). Two-hundred spermatozoa were evaluated and the spermatozoa that showed no fluorescence over the acrosome were considered to be damaged spermatozoa.

### 2.7. Statistical Analysis

The statistical analyses were performed using the SPSS 20.0 statistical software package (IBM Inc, Chicago, IL, USA). The Shapiro-Wilk test was applied to check for a normal distribution of the data. The repeated measures ANOVA or Friedman tests were used to check for differences in sperm parameters in the control group during the different times of incubation. The generalized linear model (GZLM) was performed to analyze the effects of the treatments and storage times on sperm variables. Data are shown as mean ± standard error (SE). Statistical significance was set at *p* < 0.05.

## 3. Results

### 3.1. Total Antioxidant Capacity

As shown in Table 1, the total antioxidant capacity of the CTR samples did not change during the whole incubation (*p* > 0.05). At each incubation time, there were also no differences between CTR and CTR-ox groups (*p* > 0.05). Irrespective of the incubation time, 10 mM aminoguanidine showed greater total antioxidant capacity than CTR-ox group (*p* < 0.001 at both incubation times), while 1 mM aminoguanidine showed greater total antioxidant capacity at 3.5 h of incubation only (*p* = 0.031).

Furthermore, after 2 h and 3.5 h of incubation, 10 and 1 mM aminoguanidine showed greater total antioxidant capacity than CTR samples (*p* < 0.05).

### 3.2. Sperm Motility and Kinetics

**Figure 1:**
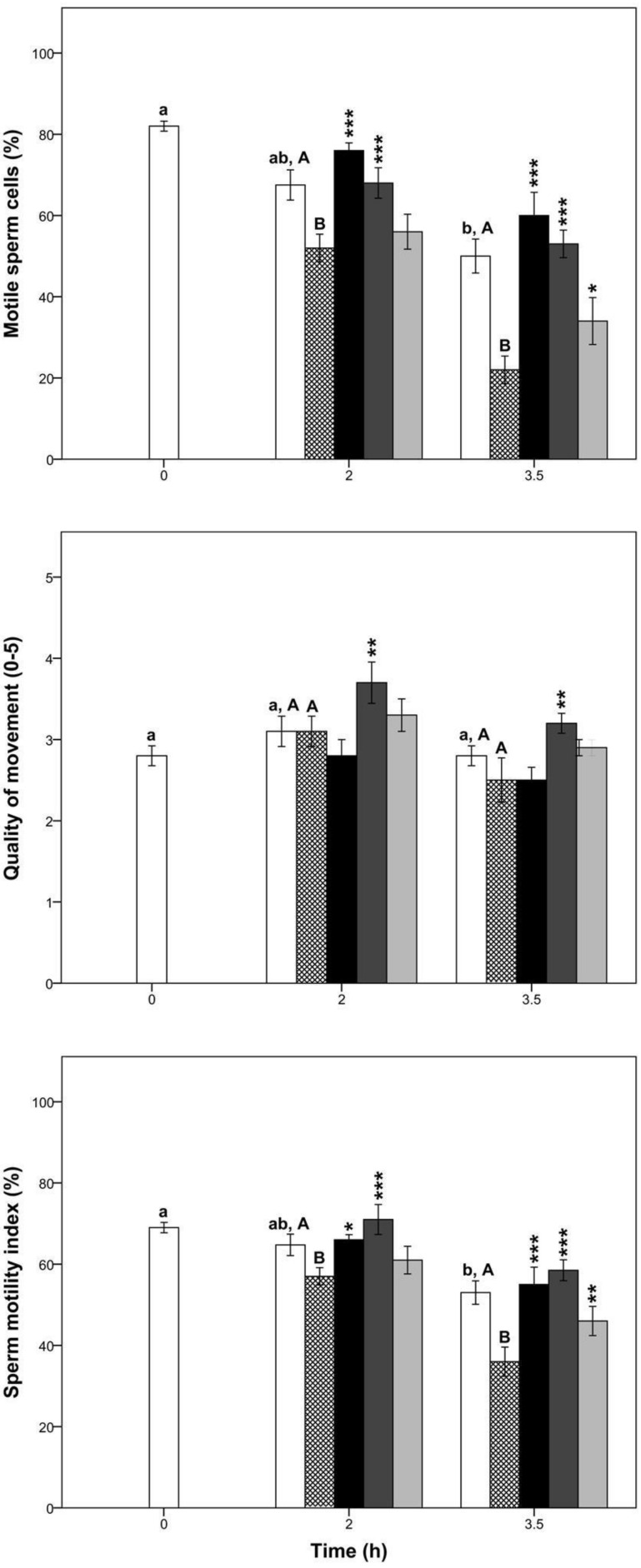
Percentage of motile sperm cells, quality of movement, and sperm motility index in boar samples submitted to oxidative stress (CTR except) and supplemented with aminoguanidine. Different superscript lower-case letters indicate significant differences (*p* < 0.05) among times for the control group without induced oxidative stress. Different superscript upper-case letters indicate significant differences (*p* < 0.05) between the control group with and without induced oxidative stress within each given time. The asterisks indicate significant differences between the treatment and the control submitted to induced oxidative stress within each given time (* *p* < 0.05; ** *p* ≤ 0.01; *** *p* ≤ 0.001). White bars = control samples; crossed bars = control samples under induced oxidative stress; black bars = 10 mM aminoguanidine; dark grey bars = 1 mM aminoguanidine; and light grey bars = 0.1 mM aminoguanidine. Data are shown as mean ± standard error of 5 replicates.

At 2 h of incubation, there were no significant differences in any sperm kinetic parameter between CTR and CTR-ox groups (*p* > 0.05, Figure 1 and Table 2). Conversely, at 3.5 h of incubation, sperm kinetic parameters (except for the BCF, LIN, and STR) were negatively affected by this ROS generator (*p* < 0.05, Figure 1 and Table 2).

Overall, our results show that aminoguanidine preserved sperm motility under oxidative stress conditions (Figure 1 and Table 2). At both times of incubation, the TM of samples that were treated with 10 and 1 mM aminoguanidine were significantly greater than those of CTR-ox group (*p* < 0.05) with a two-fold increase at 3.5 h of sperm incubation. Nevertheless, it is important to highlight that despite the great percentage of motile spermatozoa observed during the whole incubation, samples treated with 10 mM aminoguanidine tended to display a relatively non-progressive and circular movement. In this way, at 2 h of incubation, samples treated with 10 mM aminoguanidine showed a greater percentage of motile sperm cells, SMI, TM and VCL, but smaller percentage of PM, BCF, LIN, and STR compared to those of CTR-ox samples (*p* < 0.05). There was also a significant increase in the percentage of motile sperm cells, SMI, and TM at 3.5 h of incubation in samples treated with 0.1 mM aminoguanidine compared to those of CTR-ox samples (*p* < 0.05). While there were no differences in the other kinetic parameters at this aminoguanidine concentration, they tended to be greater than those of CTR-ox group (*p* > 0.05).

Interestingly, irrespective of the incubation time, there was no difference in any sperm kinetic parameter between 1 mM aminoguanidine and CTR samples (*p* > 0.05). Conversely, 10 mM aminoguanidine showed greater TM, VCL, and ALH, but smaller PM, BCF, LIN, and STR than CTR group at 2 h of incubation (*p* < 0.05). Sperm kinetic parameters in samples treated with 0.1 mM aminoguanidine did not differ or were significantly smaller than the CTR group (*p* < 0.05).

### 3.3. Lipid Peroxidation

**Figure 2:**
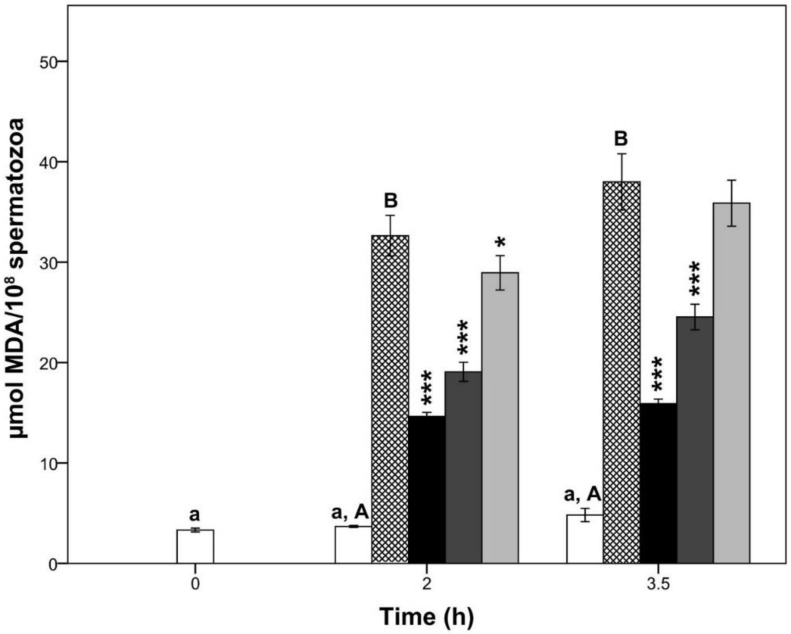
Lipid peroxidation in boar sperm samples submitted to oxidative stress (CTR except) and supplemented with aminoguanidine. Different superscript lower-case letters indicate significant differences (*p* < 0.05) among times for the control group without induced oxidative stress. Different superscript upper-case letters in the same column indicate significant differences (*p* < 0.05) between the control group with and without induced oxidative stress within each given time. The asterisks indicate significant differences between the treatment and the control submitted to induced oxidative stress within each given time (* *p* < 0.05; *** *p* ≤ 0.001). MDA = malondialdehyde; white bars = control samples; crossed bars = control samples under induced oxidative stress; black bars = 10 mM aminoguanidine; dark grey bars = 1 mM aminoguanidine; and light grey bars = 0.1 mM aminoguanidine. Data are shown as mean ± standard error of 5 replicates.

The oxidative stress induced by Fe^2+^/ascorbate provoked a significant increase in sperm lipid peroxidation at 2 h and 3.5 h of semen incubation (*p* < 0.05; Figure 2) compared to the CTR group. On the other hand, CTR samples did not change their levels of MDA during the entire period of semen incubation (*p* > 0.05). Interestingly, at 2 h of incubation, all aminoguanidine treatments showed lower levels of MDA than those of CTR-ox group (*p* < 0.05). Conversely, at 3.5 h of incubation, only 10 and 1 mM aminoguanidine showed lower levels of lipid peroxidation than CTR-ox samples (both *p* < 0.001).

As expected, all aminoguanidine treatments showed greater levels of lipid peroxidation than CTR samples (*p* < 0.001).

### 3.4. Sperm Plasma Membrane Integrity and Acrosomal Status

In general, the oxidative stress induced by Fe^2+^/ascorbate impaired the sperm plasma membrane and acrosome integrity of CTR-ox samples (Table 3). Moreover, we found that aminoguanidine protects the sperm head plasma membrane and acrosome integrity against oxidative stress (Table 3). While there were no significant differences in the sperm tail plasma membrane integrity between the values of CTR-ox samples and those of aminoguanidine treatments (*p* > 0.05), there was a trend for the latter to be greater at any concentration used.

We also found that 10 and 1 mM aminoguanidine showed a greater percentage of intact sperm head plasma membrane at 2 h (*p* = 0.025 and *p* < 0.001, respectively) and 3.5 h of sperm incubation (both *p* < 0.001) than CTR samples. Moreover, at 2 h and 3.5 h of incubation, 1 mM aminoguanidine showed a lower percentage of damaged acrosome than that of the CTR group (*p* = 0.011 and *p* < 0.001, respectively).

## 4. Discussion

The present study provides the first piece of evidence that aminoguanidine notably reduces the detrimental effects of oxidative stress in boar sperm cells in vitro. Our results clearly show that aminoguanidine has powerful antioxidant capacity, preserves the sperm motility, reduces the lipid peroxidation, and protects the plasma membrane and acrosome integrity under induced oxidative stress. Interestingly, except for the levels of MDA, sperm parameters of samples treated with 1 mM aminoguanidine did not differ or even showed better performance than those of control samples without the ROS-generating system, which demonstrates that the deleterious effects of oxidative stress were mostly abolished. As there was no cytotoxic effect shown in any sperm parameter, our results suggest that aminoguanidine could potentially be a treatment for impaired semen quality associated with high ROS levels.

Aminoguanidine is a selective inhibitor of the iNOS isoform [24], which releases large amounts of NO**^•^** and is found in spermatozoa and activated leukocytes [18,21]. In human semen, the presence of abnormal spermatozoa and activated leukocytes increases the amount of ROS over physiological levels, which causes sperm DNA damage, lipid peroxidation, and poor motility [5]. In this sense, Balercia et al. [43] found that asthenozospermic men show greater levels of NO**^•^** than normozoospermic men and that the concentration of this gasotransmitter were negatively correlated with the sperm motility. In this way, our findings indicate that aminoguanidine can be employed for protecting against the effects of oxidative stress in sperm cells, which is consistent with the findings in other cells and tissues (lung: [44]; bladder: [45]; kidney: [46]; testis: [29]). Similarly, Abbasi et al. [30,31] and Alizadeh et al. [32,33] found that the in vivo administration of aminoguanidine improves the sperm concentration, motility, viability, normal morphology, mitochondrial membrane potential, and DNA integrity in varicocelized rats where the upregulation of the iNOS isoform may lead to high levels of ROS in the semen.

As previously described in boar semen [35,47], our results confirm that Fe^2+^/ascorbate induces a state of oxidative stress characterized by increased levels of lipid peroxidation and reduced sperm motility. In addition, we also found that this ROS generator negatively affects the sperm head plasma membrane and acrosome integrity. In contrast to our findings, Guthrie and Welch [47] found that Fe^2+^/ascorbate did not affect the sperm membrane integrity (i.e., viability). This is possibly because a smaller Fe^2+^/ascorbate concentration was employed (i.e., 1 µM/30 µM) in their study. Our findings also confirm that aminoguanidine has powerful antioxidant abilities against the oxidative stress induced by Fe^2+^/ascorbate, as previously described by Yildiz et al. [25]. Irrespective of the incubation time, 10 and 1 mM aminoguanidine showed stronger antioxidant capacity than that of control samples with or without induced oxidative stress. The total antioxidant capacity of 1 mM aminoguanidine was 0.5 mM Trolox equivalents on average, which is within the range described in the boar seminal plasma [48,49]. A greater total antioxidant capacity of the seminal plasma contributes to the ability of boar sperm cells to better sustain the preservation process (liquid-storage and cryopreservation), which is also positively related to the fertility outcomes and litter size [49]. In this way, our results indicate that 1 mM aminoguanidine shows a total antioxidant capacity similar to that of boar seminal plasma, which provides further support for the beneficial effects of this compound on boar sperm parameters under induced oxidative stress.

The results of this study show that under induced oxidative stress, aminoguanidine better preserves sperm motility, plasma membrane and acrosome integrity. These three parameters are correlated with male fertility in humans [50] and other species (bulls: [51]; boars: [52]; stallions: [53]). Interestingly, at 3.5 h of incubation, 10 and 1 mM aminoguanidine showed more than twice the percentage of motile sperm cells compared to control samples under oxidative stress. Nevertheless, it is important to highlight that sperm cells treated with 10 mM aminoguanidine showed rapid curvilinear trajectories with remarkably low values of progressive and linear motility. A plausible explanation of this phenomenon might be due to the antioxidant capacity of 10 mM aminoguanidine (2 mM Trolox equivalents), which is quite above the physiological range reported in the boar seminal plasma [48,49]. As certain levels of ROS are required for a normal sperm function [8,9], 10 mM aminoguanidine may reduce the amount of ROS in such a way that it impairs some sperm kinetic parameters, but it does not affect the sperm plasma membrane and acrosome integrity. In this sense, the protective effects on the sperm plasma membrane and acrosome integrity in samples treated with 10 mM aminoguanidine were also confirmed by the lower levels of lipid peroxidation. On the other hand, 0.1 mM aminoguanidine was able to only partially prevent the damage caused by Fe^2+^/ascorbate in terms of sperm parameters. In contrast, although the sperm tail integrity tended to be greater in sperm samples supplemented with aminoguanidine, there were no differences among the latter and control group treated with Fe^2+^/ascorbate, which is likely due to the variability among replicates. The boar sperm plasma membrane shows low tolerance to the hypoosmotic conditions, which varies across breeds and between boars within the same breed [54]. In this way, despite the fact that we used pooled semen in order to reduce the male variability, factors, such as the boar and breed, may have influenced our results by increasing the variability among replicates.

Another important finding of this study is that aminoguanidine protects the acrosome integrity as shown by the two techniques employed. It is well known that acrosome integrity is a requisite for the acrosome reaction, which must occur in a timely manner in order to allow the penetration of the spermatozoon through the protective barriers of the oocyte [55]. In the porcine species, a partially induced acrosome reaction in the preincubation or fertilization media has been found to be an important cause of polyspermy, which is one major challenge in the assisted reproductive technologies of this species [56]. The exposure of boar spermatozoa to a ROS generating system triggers the acrosome reaction [57], which may lead to reduced fertilizing potential. Based on our findings, we can therefore speculate that aminoguanidine may increase the fertilization potential of porcine spermatozoa by preventing a precocious acrosome reaction under oxidative stress. Further studies, such as the in vitro and in vivo fertilizations, are needed to test our hypothesis.

In conclusion, the findings from this study demonstrate that aminoguanidine mostly abolishes the deleterious effects of oxidative stress in boar spermatozoa under in vitro conditions. Due to its antioxidant capacities, aminoguanidine preserves the boar sperm motility, reduces the lipid peroxidation, and protects the plasma membrane and acrosome integrity under oxidative stress. Interestingly, 1 mM aminoguanidine mostly eliminates the negative effects of oxidative stress as, except for the lipid peroxidation, all sperm parameters did not differ or even showed better performance than those of control samples without the ROS-generating system. As no cytotoxic effects were observed in any sperm parameters, our results suggest that aminoguanidine could be used as an effective in vitro therapeutic agent for the treatment of sperm disorders associated with oxidative stress.

## Figures and Tables

**Table 1:** Total antioxidant capacity of boar sperm samples submitted to oxidative stress (CTR except) and supplemented with aminoguanidine.

Treatment	Time (h)	Total Antioxidant Capacity (mM)
CTR	0	0.4 ± 0.1 ^a^
CTR	2	0.1 ± 0.0 ^a,A^
CTR-ox	2	0.4 ± 0.2 ^A^
Ag10-ox	2	2.1 ± 0.1 ***
Ag1-ox	2	0.5 ± 0.1
Ag0.1-ox	2	0.4 ± 0.3
CTR	3.5	0.2 ± 0.0 ^a,A^
CTR-ox	3.5	0.2 ± 0.0 ^A^
Ag10-ox	3.5	2.2 ± 0.1 ***
Ag1-ox	3.5	0.5 ± 0.1 *
Ag0.1-ox	3.5	0.2 ± 0.1

Total antioxidant capacity is expressed as Trolox equivalents. Different superscript lower-case letters indicate significant differences (*p* < 0.05) among times for the control samples without induced oxidative stress. Different superscript upper-case letters indicate significant differences (*p* < 0.05) within each given time between the control samples with and without induced oxidative stress. The asterisks indicate significant differences (* *p* < 0.05; *** *p* ≤ 0.001) within each given time between the treatments and the control samples submitted to induced oxidative stress. CTR = control; ox = samples submitted to induced oxidative stress; Ag10 = 10 mM aminoguanidine; Ag1 = 1 mM aminoguanidine; and Ag0.1 = 0.1 mM aminoguanidine. Data are shown as mean ± standard error of 5 replicates.

**Table 2:** Boar sperm kinetics in samples submitted to oxidative stress (CTR except) and supplemented with aminoguanidine.

Treatment	Time (h)	TM (%)	PM (%)	VAP (µm/s)	VCL (µm/s)	VSL (µm/s)	ALH (µm)	BCF (Hz)	LIN (%)	STR (%)
CTR	0	77.2 ± 3.5 ^a^	44.9 ± 0.9 ^a^	40.6 ± 1.3 ^a^	80.8 ± 1.9 ^a^	30.6 ± 1.0 ^a^	3.0 ± 0.1 ^a^	13.1 ± 0.2 ^a^	38.0 ± 0.9 ^a^	75.5 ± 0.8 ^a^
CTR	2	63.1 ± 6.1 ^a,A^	62.4 ± 5.6 ^b,A^	39.2 ± 2.8 ^a,A^	67.5 ± 3.2 ^b,A^	36.0 ± 2.5 ^ab,A^	2.7 ± 0.1 ^b,A^	14.4 ± 0.4 ^b,A^	51.7 ± 1.4 ^b,A^	89.7 ± 0.5 ^b,A^
CTR-ox	2	54.2 ± 4.7 ^A^	61.2 ± 5.4 ^A^	41.4 ± 3.0 ^A^	68.1 ± 5.3 ^A^	38.9 ± 2.8 ^A^	2.8 ± 0.2 ^A^	14.7 ± 0.2 ^A^	55.9 ± 1.9 ^A^	92.3 ± 0.9 ^A^
Ag10-ox	2	83.0 ± 1.6 ***	42.0 ± 2.8 ***	42.2 ± 3.3	92.9 ± 8.6 ***	30.2 ± 1.7 **	3.3 ± 0.3	12.7 ± 0.4 ***	34.2 ± 1.8 ***	70.8 ± 2.3 ***
Ag1-ox	2	65.6 ± 1.4 *	63.2 ± 2.6	45.0 ± 4.9	79.6 ± 9.4	40.7 ± 4.1	3.1 ± 0.4	14.0 ± 0.4	50.8 ± 2.3 *	88.6 ± 1.4 *
Ag0.1-ox	2	56.7 ± 4.7	58.7 ± 3.1	39.4 ± 4.0	68.4 ± 9.0	35.8 ± 3.1	2.7 ± 0.3	14.4 ± 0.4	52.5 ± 3	89.5 ± 2.2
CTR	3.5	60.0 ± 6.3 ^a,A^	62.8 ± 6.0 ^b,A^	41.5 ± 3.3 ^a,A^	68.9 ± 4.2 ^b,A^	38.6 ± 3.3 ^b,A^	2.7 ± 0.2 ^ab,A^	14.8 ± 0.4 ^b,A^	54.6 ± 1.9 ^b,A^	90.9 ± 1.0 ^b,A^
CTR-ox	3.5	24.9 ± 4.4 ^B^	43.7 ± 8.0 ^B^	29.4 ± 1.4 ^B^	45.5 ± 2.9 ^B^	27.9 ± 1.4 ^B^	1.9 ± 0.1 ^B^	15.1 ± 0.4 ^A^	62.3 ± 2.4 ^B^	93.6 ± 1.4 ^A^
Ag10-ox	3.5	69.0 ± 5.6 ***	52.6 ± 4.0	36.0 ± 3.2	78.1 ± 6.6 ***	29.3 ± 2.5	2.8 ± 0.2 ***	12.9 ± 0.4 ***	39.4 ± 1.6 ***	80.5 ± 1.7 ***
Ag1-ox	3.5	59.0 ± 3.8 ***	63.8 ± 3.3 ***	40.4 ± 2.5 **	69.2 ± 3.1 **	37.7 ± 2.5 **	2.8 ± 0.1 **	14.2 ± 0.4	54.1 ± 1.2 ***	91.6 ± 0.6
Ag0.1-ox	3.5	39.6 ± 4.8 *	53.1 ± 5.5	32.7 ± 1.9	53.0 ± 5.0	30.8 ± 1.5	2.3 ± 0.2	15.0 ± 0.4	59.1 ± 2.5	93.0 ± 0.8

Different superscript lower-case letters in the same column indicate significant differences (*p* < 0.05) among times for the control group without induced oxidative stress. Different superscript upper-case letters in the same column indicate significant differences (*p* < 0.05) between the control group with and without induced oxidative stress within each given time. The asterisks indicate significant differences between the treatment and the control submitted to induced oxidative stress within each given time (* *p* < 0.05; ** *p* ≤ 0.01; *** *p* ≤ 0.001). TM = total motility; PM = progressive motility; VAP = average path velocity; VCL = curvilinear velocity; VSL = straight-line velocity; ALH = amplitude of lateral head displacement; BCF = beat-cross frequency; LIN = linearity; STR = straightness; CTR = control; ox = samples submitted to induced oxidative stress; Ag10 = 10 mM aminoguanidine; Ag1 = 1 mM aminoguanidine; and Ag0.1 = 0.1 mM aminoguanidine. Data are shown as mean ± standard error of 5 replicates.

**Table 3:** Boar sperm plasma membrane integrity and acrosomal status in samples submitted to oxidative stress (CTR except) and supplemented with aminoguanidine.

Treatment	Time (h)	Intact Head Plasma Membrane (%)	Intact Tail Plasma Membrane (%)	Normal Apical Ridge (%)	Damaged Acrosome (%)
CTR	0	83.2 ± 0.6 ^a^	27.9 ± 2.5 ^a^	94.5 ± 0.3 ^a^	2.1 ± 0.2 ^a^
CTR	2	76.4 ± 0.5 ^b,A^	23.0 ± 4.6 ^ab,A^	92.1 ± 0.5 ^b,A^	3.2 ± 0.4 ^b,A^
CTR-ox	2	71.9 ± 0.7 ^B^	16.3 ± 4.5 ^A^	87.6 ± 1.3 ^B^	3.9 ± 0.4 ^A^
Ag10-ox	2	78.8 ± 0.6 ***	24.3 ± 4.3	93.1 ± 0.5 ***	2.7 ± 0.3 **
Ag1-ox	2	81.6 ± 1.0 ***	20.2 ± 3.8	91.4 ± 1.1 ***	2.2 ± 0.2 ***
Ag0.1-ox	2	74.3 ± 0.3 *	21.0 ± 4.7	91.1 ± 0.5 ***	3.3 ± 0.4
CTR	3.5	69.3 ± 1.2 ^c,A^	20.4 ± 4.0 ^b,A^	88.7 ± 0.8 ^c,A^	4.7 ± 0.3 ^c,A^
CTR-ox	3.5	62.8 ± 0.8 ^B^	14.1 ± 3.7 ^A^	84.9 ± 1.0 ^B^	6.2 ± 0.3 ^B^
Ag10-ox	3.5	73.5 ± 0.8 ***	22.0 ± 3.9	90.3 ± 1.0 ***	4.2 ± 0.4 ***
Ag1-ox	3.5	78.3 ± 1.1 ***	18.1 ± 4.5	90.6 ± 1.1 ***	3.1 ± 0.2 ***
Ag0.1-ox	3.5	67.7 ± 1.2 ***	16.0 ± 4.61	90.5 ± 0.6 ***	5.3 ± 0.3 *

Different superscript lower-case letters in the same column indicate significant differences (*p* < 0.05) among times for the control group without induced oxidative stress. Different superscript upper-case letters in the same column indicate significant differences (*p* < 0.05) between the control group with and without induced oxidative stress within each given time. The asterisks indicate significant differences between the treatment and the control submitted to induced oxidative stress within each given time (* *p* < 0.05; ** *p* ≤ 0.01; *** *p* ≤ 0.001). CTR = control; ox = samples submitted to induced oxidative stress; Ag10 = 10 mM aminoguanidine; Ag1 = 1 mM aminoguanidine; and Ag0.1 = 0.1 mM aminoguanidine. Data are shown as mean ± standard error of 5 replicates.

## Supplementary Materials

The following are available online at http://www.mdpi.com/1999-4923/10/4/212/s1, Supplementary Dataset 1. Dataset of boar sperm parameters under induced oxidative stress (CTR except) and supplemented with aminoguanidine.

## Availability of Materials and Data

All data generated or analyzed during this study are included in this article and Supplementary Information file.
